# Tubomanometry and Symptom Outcomes in Eustachian Tube Dysfunction Associated with Chronic Nasal Disease

**DOI:** 10.3390/audiolres16030070

**Published:** 2026-05-10

**Authors:** Sofia Anastasiadou, Petros Karkos, Jannis Constantinidis, George Psillas

**Affiliations:** 1st Academic ENT Department, Aristotle University of Thessaloniki, AHEPA Hospital, No. 1, Stilponos Kyriakidi St., 546 36 Thessaloniki, Greecepsill@otenet.gr (G.P.)

**Keywords:** Eustachian tube dysfunction, tubomanometry, chronic nasal disease, chronic rhinitis, nasal septal deviation, chronic rhinosinusitis with nasal polyps, nasal irrigation, septoplasty, functional endoscopic sinus surgery, symptom improvement

## Abstract

**Background**: Eustachian Tube Dysfunction (ETD) presents significant diagnostic challenges, particularly in patients with chronic nasal disease, which often mimic or complicate ETD symptoms. Tubomanometry (TMM) is emerging as an objective tool for diagnosing ETD, but its application in patients with concurrent nasal pathologies remains underexplored. **Methods**: One hundred patients with concurrent ETD and chronic nasal disease were recruited. They were categorized into three groups: chronic rhinitis (35 patients, group A), nasal septal deviation (31 patients, group B) and chronic rhinosinusitis with polyps (34 patients, group C). Treatments included nasal irrigation, septoplasty, and nasal polypectomy with functional endoscopic sinus surgery, respectively. The TMM parameters (R, C2 and C2–C1 scores) were assessed before and after interventions. The patient reported outcome measures (ETDQ-7 and NOSE scores) were also recorded and statistically correlated with TMM measures. **Results**: Group A showed improvements in R, C2 and C2–C1 scores and mild post-treatment reductions in ETDQ-7 and NOSE scores. Similar improvements were observed in Group B, with significant symptom reduction post-septoplasty, particularly on the side of the nasal septal deviation. Group C demonstrated the greatest improvement, with significant improvements in TMM values and substantial reductions in both ETDQ-7 and NOSE scores. The statistical results revealed correlations between the treatment of nasal pathologies and ETDQ-7 and NOSE scores. **Conclusions**: All TMM parameters improved in each group following the nasal intervention. This study highlights the utility of TMM in evaluating ETD in the context of chronic nasal disease and suggests that treating underlying nasal conditions can significantly alleviate ETD symptoms.

## 1. Introduction

Eustachian Tube Dysfunction (ETD) presents a complex and nuanced challenge in otolaryngology, making difficult to fully understand its underlying mechanisms and establish clear diagnostic standards. The presence of concurrent chronic nasal disease further complicates the clinical scenario by introducing additional factors that can mimic ETD symptoms. Traditionally, patient-reported outcome measures (PROMs) are used to assess the severity of ETD. Tubomanometry (TMM) is gaining recognition as a promising objective diagnostic tool for ETD, where available [1,2,3]. However, the application of TMM in the presence of chronic nasal disease remains largely unexplored. Additionally, the optimal sequence of addressing nasal pathology and ETD, or whether simultaneous interventions should be considered, has not been thoroughly examined in the literature. There are limited number of studies discussing this complex relationship and how one condition may influence the other [4,5]. The current literature typically assesses post-operative outcomes using PROMs, rather than objective measurements, such as TMM values [6,7,8,9]. This study aims to explore the utility of TMM both before and after the treatment of chronic nasal disease in patients with pre-existing ETD.

Chronic nasal disease is a multifaceted condition, most commonly manifesting as chronic rhinitis (CR) either allergic or non-allergic, nasal septal deviation (NSD), or chronic rhinosinusitis with (CRSwP) or without polyps. Often, multiple pathologies coexist, involving both anatomical and inflammatory changes that contribute to chronic nasal conditions. Discriminating between these conditions can be challenging, which adds to the complexity of understanding the relationship between ETD and chronic nasal disease. Furthermore, treatment options, such as nasal irrigation with normal saline, intranasal steroid sprays, nasal septoplasty, nasal polypectomy and functional endoscopic sinus surgery (FESS) are tailored to each patient to address their specific pathology, though these treatments are frequently combined to target multiple symptoms. This study aims to investigate whether treating specific chronic nasal pathologies can simultaneously improve ETD symptoms.

## 2. Materials and Methods

**Figure 1:**
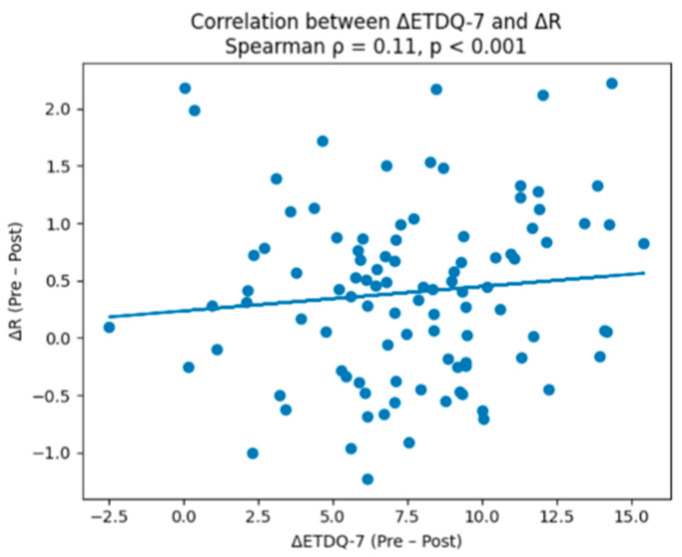
ET-specific symptom improvement (ΔETDQ-7) correlating with objective ET function (ΔR). The line slopes upward because ΔETDQ-7 and ΔR are positively correlated (ρ = 0.52), meaning that greater improvement in ET–specific symptoms correspond to greater improvement in objective tube opening function.

The total number of patients recruited was 100, and they were subsequently categorized into three distinct groups, based on their chronic nasal pathology and received interventions tailored to their specific condition (Figure 1): Group A (chronic rhinitis) included 35 patients suffering from nasal congestion and discharge. On examination, they presented nasal mucosal changes with irritation, inflammation and clear discharge, associated with inferior turbinate hypertrophy; no prominent nasal septal deviation or polyposis was detected. Their treatment consisted of steroid nasal spray and topical saline irrigation. Group B (nasal septal deviation—NSD) included 31 patients with nasal septal deviation and nasal obstruction, who underwent septoplasty under general anesthesia; no other nasal pathology was observed, such as polyposis, turbinate hypertrophy or allergy. It is common knowledge that compensatory inferior turbinate hypertrophy commonly accompanies septal deviation. In our cohort, patients with significant turbinate hypertrophy requiring additional surgical intervention were excluded, as the study aimed to evaluate the effect of septal correction on Eustachian tube function as an isolated factor. Mild compensatory turbinate enlargement may have been present but was not considered clinically significant or requiring treatment. Therefore, for subgroup analysis within the NSD group, it was clearly documented where the deviation of the septum and all patients with concurrent polyposis were excluded for the group. Group C (CRSwNP) included 34 patients diagnosed with chronic rhinosinusitis with bilateral nasal polyps primarily occupying the middle meatus. All patients underwent standardized surgical management consisting of endoscopic nasal polypectomy combined with functional endoscopic sinus surgery (FESS). The distribution and extent of polyps within the nasal cavity were documented to ensure cohort consistency; however, detailed endoscopic grading was not systematically collected, and the extent of surgery was tailored to individual anatomical and disease considerations. This approach allowed assessment of Eustachian tube function following surgical intervention while reflecting real-world clinical practice. Demographics of the three groups are presented in Table 1.

Information leaflets were provided to potential participants, followed by individual meetings with researchers to explain the study procedures. All participants gave informed consent after a thorough discussion of the study details. The study protocol was approved by the Committee of Bioethics and Deontology at the Hospital.

### 2.1. Tubomanometry

Tubomanometry (TMM) was performed to objectively assess Eustachian tube dysfunction (ETD) and quantify the severity of functional impairment. This technique measures pressure changes in the nasopharynx during voluntary swallowing, providing physiological insight into Eustachian tube opening and pressure transmission. The R-value, derived from TMM, serves as an index of ET function: an R-value ≤ 1 indicates normal, timely tube opening, whereas an R-value > 1 is considered pathological, reflecting delayed or impaired opening. Additional parameters include C1, expressed in millibars (mbar), which represents the pressure threshold at which nasopharyngeal pressure begins to rise, and C2, also in mbar, corresponding to the maximum pressure achieved in the nasopharynx during measurement. The difference between C2 and C1 (C2–C1, in seconds) reflects the time required for the nasopharyngeal pressure to reach its peak, providing a dynamic measure of tube compliance and opening efficiency. Together, these parameters allow comprehensive evaluation of both the timing and magnitude of Eustachian tube function, complementing subjective symptom assessments such as the ETDQ-7 [10]. All TMM machines work within a distance from the sea in terms of pressure and metrics. We standardized our specific metrics testing 50 healthy individuals before the start of the study. This allowed our study to present reliable results. A control group was not originally approved by the Hospital Ethics Committee due to potentially not providing appropriate treatment to unhealthy individuals in need.

### 2.2. Patient Reported Outcome Measures

The patient reported outcome measures (PROMs) were used to subjectively evaluate the symptoms of ETD and chronic nasal disease; for this, the Eustachian Tube Dysfunction Questionnaire-7 (ETDQ-7) and the Nasal Obstruction Symptom Evaluation (NOSE) questionnaire were selected, respectively [11,12]. As per the literature findings, scores were considered pathological when they were greater than 14,5 for ETDQ-7 and more than 25 for NOSE.

### 2.3. Inclusion Criteria

Inclusion criteria focused on specific nasal pathology corresponding to one of the three groups (A–C), objective findings of ETD (either retracted tympanic membrane or Type C tympanogram), age over 18 years and symptoms of ETD lasting more than 12 weeks; moreover, the PROMs’s (ETDQ-7, NOSE) scores should be pathological (ETDQ-7 > 14.5, NOSE > 25) to demonstrate concurrent nasal disease and Eustachian tube dysfunction.

### 2.4. Exclusion Criteria

The exclusion criteria comprised previous ENT operations, severe ear pathology, adenoid hypertrophy, post nasal space lesions, previous head and neck radiotherapy, congenital abnormalities, and temporomandibular joint dysfunction.

The TMM was applied to all groups with specific nasal pathologies as well as PROMs (ETDQ-7 and NOSE scores) were given to all patients before and after treatment. The patients in each group were re-assessed by the research team six months post completion of each treatment modality. This was considered adequate time for the nasal structures and mucosa to recover and allowed patients to have an objective idea on how their symptoms have changed. An assessment closer to the operation date was considered not representative due to the recovery process and the possible effect on the measurements. The patients were asked to complete the NOSE and ETDQ-7 questionnaires and underwent examination and TMM to compare the pre and post treatment results.

### 2.5. Statistical Analysis

Data analysis was performed using SPSS Statistics software version 28 (IBM Corp., New York, NY, USA). The Spearman rank correlation coefficient, a nonparametric measure, was used to assess the strength and direction of the relationship between NOSE and ETDQ-7 scores with R, C2, and C2–C1. Values close to 1 indicate a strong positive correlation between the compared values, while values close to −1 indicate a strong negative correlation. 

To assess the relationship between objective ET function and subjective symptom scores, Spearman rank correlation coefficients (ρ) were calculated between the ETDQ-7 and NOSE scores and tubomanometry (TMM) parameters (R, C2, and C2–C1) at pre-treatment, post-treatment, and for the change (Δ) values. A two-tailed Spearman rank correlation test was further performed to investigate statistically significant interactions between variables. Comparisons between groups were statistically analyzed using the Kruskal–Wallis test. Dunn’s post hoc test was used to conduct pairwise comparisons between groups after a significant result was found with the Kruskal–Wallis test. A *p*-value < 0.05 was considered indicative of statistical significance.

## 3. Results

We hypothesize that improving nasal airway patency—whether through medical or surgical intervention—enhances pressure transmission to the nasopharynx, facilitates Eustachian tube opening, and leads to measurable improvements in tubomanometry parameters. This effect is influenced by key physiological mechanisms, including nasal airflow, mucosal inflammation, and obstruction at the nasopharyngeal orifice. Structural factors, such as septal deviation or nasal polyps, may further impact airflow dynamics and nasopharyngeal pressure regulation. The following results examine the outcomes of these interventions across the patient groups in this context.

In terms of demographics, in Group A, females constituted the majority of participants (60%), while Group C similarly demonstrated a female predominance (55.9%) (Table 1). In contrast, Group B included a higher proportion of male participants (58.1%). Overall, all groups included both sexes with a relatively balanced gender distribution. Hence, any sex distribution differences likely reflect the clinical characteristics of the recruited population rather than a pathophysiological factor influencing the results.

Regarding age, clear differences were observed among the groups. Group B had the lowest mean age (36.0 ± 9.09 years), indicating a younger study population, whereas Group A showed an intermediate mean age of 41.2 ± 14.8 years. Group C consisted of the oldest participants, with a mean age of 48.0 ± 12.3 years. The age distributions suggest distinct demographic profiles across the three groups, which align with the stratification of the study cohorts.

### 3.1. Group A: Chronic Rhinitis

In group A, the mean pre-intervention ETDQ-7 and NOSE scores were 21.98 and 33.85, respectively; after the intervention, scores decreased to 13.93 and 18.85, showing significant improvement (*p* < 0.05) (Table 2).

Pre-intervention R-scores (1.15 right, 1.23 left) indicated ETD (R > 1). During the post-treatment period, the R-score decreased significantly on both sides (*p* < 0.05), reflecting clear improvement. The C2 scores slightly increased, likely due to reduced nasal mucosal inflammation, which allowed for more airflow into the nasal cavity. This resulted in increased pressures into the nasopharynx demonstrated by elevated C2 scores. The C2–C1 values showed a small, non-statistically-significant decrease.

### 3.2. Group B: Nasal Septal Deviation

Preoperatively (Table 2), group B exhibited the highest mean ETDQ-7 score (23.17) and the lowest C2 scores on both sides compared to the other groups. Group B showed the most significant improvement in ETDQ-7 scores among all groups (*p* < 0.01). Preoperatively, the mean R values were pathological (R > 1) on both sides; postoperatively, they decreased to 0.68 on the right side and to 0.96 on the left side, indicating objective improvement. The improvement on the right side was statistically more significant (*p* < 0.01) compared to left, likely due to the frequent presence of a septal spur on the right side (85%). The incidental presence of the nasal septal deviation on the right side for most patients was already highlighted during recruitment period and was documented for each patient in the group. Postoperatively, the mean C2 values increased on both sides (*p* < 0.05), due to improved nasal airflow and pressure transmission. The mean C2–C1 values also decreased on both sides, most notably on the right (*p* < 0.05), suggesting faster ET opening after surgery.

### 3.3. Group C: Chronic Rhinosinusitis with Polyps

The NOSE score and the C2–C1 were higher scores compared to the other groups, indicating the negative effect of the disease on nasal permeability. Post-treatment data (Table 2) showed significant improvement in the ETDQ-7 (*p* < 0.05) and NOSE (*p* < 0.01) mean scores. The NOSE mean score decreased more significantly in this group compared to groups A and B (*p* < 0.01). Additionally, all TMM parameters in group C, such as the R, C2 and C2–C1-scores, showed significant improvement on both sides (*p* < 0.01), indicating enhanced ET function due to increased nasopharyngeal air passage after nasal polyp removal.

### 3.4. Correlation Between and TMM and PROMs Parameters

**Figure 2:**
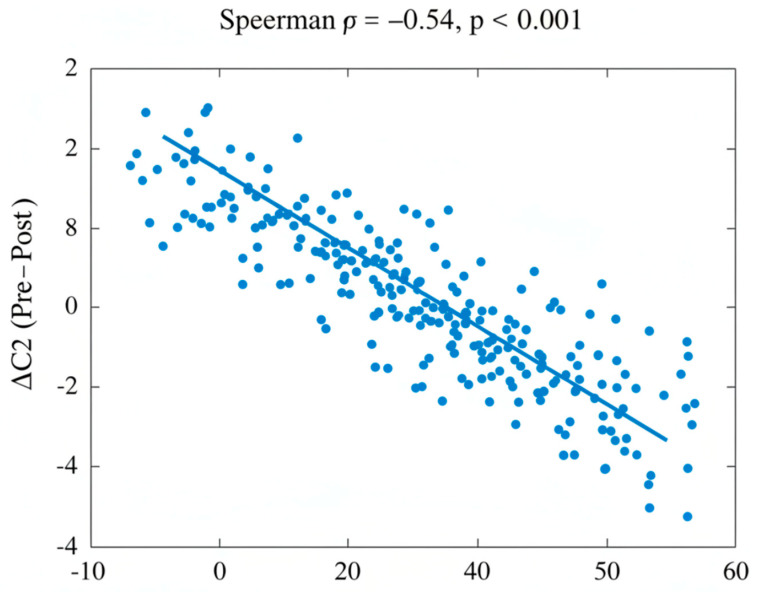
Nasal symptom improvement (ΔNOSE) correlating with ΔC2. slopes downward because ΔNOSE and ΔC2 are negatively correlated (ρ = −0.49); as nasal obstruction symptoms improve, the maximal air pressure C2 in nasopharynx becomes greater, resulting in better tube opening function.

Spearman rank correlation analysis demonstrated significant associations between subjective symptom improvement and objective ET function. Across the pooled cohort (100 patients), pre-treatment ETDQ-7 scores were moderately positively correlated with R values and C2–C1 times (Table 3), and negatively correlated with C2, indicating that worse reported symptoms corresponded to delayed tube opening and lower nasopharyngeal pressures. Post-treatment ETDQ-7 scores remained significantly correlated with R (Figure 1), C2–C1, and C2. Analysis of the change (Δ) values revealed that greater reductions in ETDQ-7 scores were associated with greater improvements in TMM parameters: ΔR (ρ = 0.52, *p* < 0.001), ΔC2–C1, and ΔC2. Similarly, NOSE scores showed significant correlations with TMM parameters, with ΔNOSE correlated with ΔR, ΔC2–C1, and ΔC2 (Figure 2). These findings indicate a moderate-to-strong correspondence between improvements in patient-reported symptoms and objective ET function, confirming that TMM reliably reflects clinical changes following treatment of chronic nasal pathologies.

## 4. Discussion

Chronic nasal disorders, marked by ongoing inflammation and structural variations, can significantly affect the functioning of the ET. In cases of CR, mucosal inflammation and increased nasal secretions can impair the openness of the ET, leading to symptoms of ETD. The inflammatory process might spread to the ET orifice or simply nasal obstruction might affect airflow in the nasopharynx and alter pressures in the ET. ETD may occur independently of allergic background, and that chronic mucosal inflammation itself may impair Eustachian tube function. Also, NSD can impact nasal airflow dynamics, altering the pressure conditions within the ET. A sizeable septal spur or a severe deviation might completely obstruct one nostril and therefore inflow of air in the nasopharynx and ET orifice [13]. Additionally, CRSwNP adds complexity by causing inflammation and potential blockages in the nasal and sinus areas [14]. As of now, no studies in the existing literature have explored the correlation between various nasal pathologies and compared their effects on objective TMM assessments and patient-reported outcome measures (PROMs), such as the ETDQ-7 and NOSE questionnaires, before and after treatment modalities.

The relationship between nasal airflow and Eustachian tube function is critical, as adequate airflow helps maintain normal pressure and facilitates tube opening. The role of mucosal inflammation and obstruction at the nasopharyngeal orifice further influences tube function by increasing resistance and impairing mucociliary clearance. Additionally, the effect of septal deviation and nasal polyposis on airflow dynamics and pressure regulation can exacerbate Eustachian tube dysfunction, demonstrating how both structural and inflammatory factors interact to impact middle ear ventilation.

In our study, the pre-treatment R values, reflecting tube opening latency, were abnormally high in all groups, demonstrating impaired or delayed ET function. In patients in group A, the abnormal R was attributed to persistent mucosal inflammation and excess secretions, which compromised the ET lumen and ventilation. In CR, the high prevalence of dysfunction may be explained by mucosal oedema extending toward the nasopharyngeal orifice, mechanical obstruction by secretions, and compensatory hypersecretion within the tube [15]. Persistent inflammation may create a feedback loop, promoting allergen retention and further impairing tube function, consistent with clinical and animal model studies demonstrating histamine-mediated mucosal swelling and ciliary dysfunction [16]. According to studies [17,18,19], there is a high correlation between allergic rhinitis and ETD, especially in pediatric populations and therefore tympanometry was used to assess severity of the disease. A pathophysiological relation between CR, especially allergic rhinitis and otitis media has been advocated [20]. In this context, Ma et al. [16] performed TMM in house dust mite allergy patients where ETD appears to be more frequent due to exposure of the ET orifice to the same allergens affecting the nasal mucosa. However, in other studies, this ETD appears independent of allergic background, suggesting that even non-allergic chronic rhinitis can substantially impair ET performance [21,22]. Adams et al. supported no correlation between the two pathologies; hence some patients experience persistence of ETD symptoms despite rhinitis treatment [23]. In our study, treatment of CR with nasal irrigation and a steroid nasal spray appears to improve objective and subjective ETD measurements with PROMs and TMM values to reflect this significant improvement.

Patients in group B demonstrated the worst pre-treatment score on the ETDQ-7 questionnaire compared to the other groups. The septal deviation may alter intranasal airflow dynamics, which can further exacerbate ETD. This was observed in our patients, where the C2 value was abnormally low due to reduced air pressure in the nasopharynx. Septal spurs create turbulent airflow and localized dryness, which may reduce mucociliary clearance and disrupt normal pressure regulation [24,25]. The impact is particularly pronounced on the side of the septal spur, aligning with observations that septal deviation is frequently associated with ipsilateral ETD [14]. This highlights the critical role of anatomical factors in predisposing to functional impairment.

Chronic rhinosinusitis with nasal polyps (group C) involves both inflammatory and mechanical mechanisms, resulting in mucosal oedema and direct obstruction at the middle meatus and nasopharyngeal interface. During the pre-treatment period, this led to abnormally high NOSE and C2–C1 scores. A prolonged C2–C1 time may suggest a slower pressure increase in the nasal and nasopharyngeal cavity, potentially indicating worse ET function. These findings demonstrate that polyps not only contribute to nasal obstruction but also significantly exacerbate ETD, highlighting the importance of addressing both components in clinical management.

Following intervention, objective and subjective measures revealed significant improvements across all patient groups, with patterns reflecting disease-specific mechanisms. Septoplasty resulted in the greatest reduction in ETD symptoms confirming that correcting anatomical obstruction directly enhances tube function. Moreover, TMM parameters, such as C2 and C2–C1 were significantly improved particularly on the side of the septal spur, confirming the relief of the mechanical obstacle in the nasal cavity. Emerging evidence in the current literature highlights the impact of NSD and septoplasty on ET function. Kaya et al. [26] assessed the effect of septoplasty on ETD and identified significant improvements. Other studies advocate for synchronous septoplasty and tympanoplasty, acknowledging the potential effect of nasal pathology on ET function [27]. However, there is no direct evidence on the relationship between NSD and ETD, with most studies relying on questionnaires and traditional examination methods, such as tympanometry, to confirm the presence of ETD. Exploring the Lebanese population, Al Karaki et al. [28] found that the side of ETD correlated with the location of NSD, which is consistent with the findings in this study. Lima et al. [13] recently suggested that NSD exacerbates ETD symptoms, and that septoplasty might alleviate the condition, based on ETDQ-7, similarly to Daum et al. [27] study on NSD and ETD. Overall, our study’s results support current literature findings. However, we used TMM scores (rather than solely relying on PROMs) to maximize the reliability and objectivity of the post-operative results. We acknowledge that TMM is not widely available, and it is not always possible to assess ETD severity using the tubomanometer; therefore, tympanometry and PROMs are often used as alternatives.

Functional endoscopic sinus surgery with polyp removal produced the largest improvement in nasal obstruction, as measured by NOSE scores, and also yielded substantial gains in ET function. In our study, all TMM parameters in group C, such as the R, C2 and C2–C1 scores, showed significant improvement on both sides, suggesting enhanced ET function due to increased nasopharyngeal air passage following nasal polyp removal. These results certified that relieving both mechanical and inflammatory barriers facilitates optimal ventilation and symptom relief. Similar findings have been reported in recent studies correlating CRSwNP with ETD. While some papers report mixed results on the management of ETD with medical treatment for CRS [15,29,30], the majority support surgical treatment of CRS as an effective means to improve ETD symptoms simultaneously [31]. Most recent studies relied on PROMs and tympanometry as their main outcome measures, given that TMM is not widely available for clinical practice or research [32,33,34]. These studies support our findings, though they primarily utilized commonly used diagnostic modalities and quality of life measurement tools [8,35]. Vandersteen et al. [14] demonstrated that TMM can effectively identify ETD improvement after FESS for nasal polyps, similar to our study’s results. Regarding the combination of FESS and ET balloon tuboplasty, there is increasing interest in monitoring results of two synchronous procedures to treat both conditions. Hsieh et al. [36] recently published a randomized controlled trial supporting the combination of ET balloon tuboplasty and FESS to treat CRS and ETD in patients suffered from both conditions. These studies further support the strong correlation between ETD and CRS. However, to our knowledge, there is no Level I evidence proving this relationship at present.

Medical management of chronic rhinitis, consisting of intranasal corticosteroids and saline irrigations, led to moderate improvements. While R values decreased and nasopharyngeal pressure dynamics (C2) improved, the magnitude of change was less pronounced than with surgical interventions, reflecting the primarily inflammatory, rather than structural, etiology. Evidence from the literature on medical management of ETD is mixed; some studies demonstrate symptomatic improvement with intranasal steroids, while systematic reviews highlight the lack of high-quality, reproducible data [37,38,39].

Overall, our study demonstrated that chronic nasal pathologies exert differential effects on ET function. Septal deviation primarily affects ETD through anatomical obstruction and altered airflow dynamics, chronic rhinitis influences ET function via mucosal inflammation and secretory changes, and chronic rhinosinusitis with polyps represents a combination of mechanical and inflammatory insults. The degree of improvement followed this order: FESS with polyp removal, septoplasty, and medical management of chronic rhinitis. In patients undergoing FESS, the combination of reduced inflammation and removal of obstructive tissue led to the most substantial functional recovery. Septoplasty predominantly improved function ipsilateral to the septal spur, while medical therapy yielded modest gains across both nasal cavities. These objective improvements were consistent with subjective questionnaire findings, confirming the utility of ET manometry as a sensitive tool for assessing therapeutic efficacy.

These findings have direct clinical implications. Preoperative evaluation of ET function can help predict postoperative outcomes, guide surgical planning, and identify patients who are likely to benefit most from targeted therapy. Moreover, the parallel improvement in manometric parameters and patient-reported outcomes supports the integration of objective assessments alongside with clinical evaluation to provide a comprehensive understanding of disease impact and treatment efficacy. Ultimately, recognizing the interplay between structural, inflammatory, and mechanical factors enables clinicians to optimize care, improve patient quality of life, and reduce the long-term consequences of chronic ETD.

### 4.1. Future Prospects

The findings of this study inspire further exploration of TMM as a critical diagnostic and monitoring tool in the management of ETD, particularly in patients with concurrent chronic nasal disease. Future research could focus on larger, multi-center studies to validate TMM’s efficacy across diverse populations and various types of nasal pathology. Additionally, longitudinal studies are needed to assess the long-term benefits of TMM in tracking ETD symptoms over time, particularly following various therapeutic interventions.

Another promising future application is the integration of TMM with advanced imaging techniques and emerging biomarkers, which could enhance the understanding of ETD pathophysiology and lead to more tailored therapeutic approaches. Moreover, combining procedures such as endoscopic nasal operations with balloon dilation of the ET is likely to attract future research interest. Further exploration of TMM’s potential in pediatric populations and in cases of recurrent ETD may yield important insights, potentially broadening its applicability in clinical practice.

### 4.2. Limitations

Despite the promising results, this study has several limitations that must be acknowledged. First, the absence of a control group limits the ability to establish a causal relationship between treatment of nasal pathology and improvements in Eustachian tube function. Second, the sample size of 100 patients, while sufficient for preliminary analysis, may limit the generalizability of the findings, and larger multicenter studies are needed to confirm these results. Third, the study included a heterogeneous patient population with varying underlying nasal pathologies, and detailed subclassification—such as allergic versus non-allergic chronic rhinitis or grading of CRSwNP—was not systematically performed. There was more focus on ETD and the intervention effect on ET function rather than nasal pathology itself. Fourth, while surgical interventions were performed according to standard clinical practice, variations in technique and the presence of mild compensatory turbinate hypertrophy may have introduced confounding effects. Finally, although tubomanometry provides objective measurements, the use of department-specific equipment and reliance on self-reported symptom scores introduces potential variability and subjectivity. Future prospective studies with control groups, standardized protocols, larger sample sizes, and longer follow-up are warranted to validate and expand upon these findings.

## 5. Conclusions

This study highlights the utility of tubomanometry as a diagnostic and monitoring tool for Eustachian tube dysfunction (ETD) in patients with chronic nasal disease. When combined with patient-reported outcomes (ETDQ-7, NOSE), tubomanometry effectively identified ETD in chronic rhinitis, nasal septal deviation, and chronic rhinosinusitis with polyps, both before and after treatment. Improvements in tubomanometry parameters closely mirrored subjective symptom relief, with the greatest post-treatment gains observed in CRSwNP (Group C), followed by septal deviation (Group B) and chronic rhinitis (Group A). Importantly, tubomanometry demonstrated consistent diagnostic performance across different nasal pathologies and objectively tracked ETD improvements after both medical and surgical interventions, emphasizing the role of nasal pathology management in ETD treatment.

## Figures and Tables

**Table 1:** Patient characteristics for each group.

		Group A	Group B	Group C
Gender	M	40% (*n* = 14)	58.1% (*n* = 18)	41.1% (*n* = 15)
	F	60% (*n* = 21)	41.9% (*n* = 13)	55.9% (*n* = 19)
Age	Mean (SD)	41.2 (14.8)	36 (9.09)	48 (12.3)

**Table 2:** ETDQ-7, NOSE, RR and LR values among groups.

		Group A	Group B	Group C
ETDQ-7	Pre-operative	21.98	23.17	19.95
	Post-operative	13.93	12.6	13.16
NOSE	Pre-operative	33.85	62.4	80.15
	Post-operative	18.85	30.6	18.95
R-score (right side)	Pre-operative	1.15	1.84	2.01
	Post-operative	0.76	0.68	0.78
R-score (left-side)	Pre-operative	1.23	1.58	1.99
	Post-operative	0.76	0.96	0.85
C2-score (right-side)	Pre-operative	26.25	22.55	22.80
	Post-operative	31.83	29.75	35.90
C2-score (left-side)	Pre-operative	28.74	26.07	27.13
	Post-operative	29.30	31.63	36.81
C2–C1-score (right-side)	Pre-operative	0.28	0.56	0.65
	Post-operative	0.26	0.54	0.34
C2–C1-score (left-side)	Pre-operative	0.31	0.32	0.71
	Post-operative	0.29	0.3	0.38

**Table 3:** Correlation between TMM and PROMs values.

Variable Pair	Pre-Treatment ρ	*p*-Value	Post-Treatment ρ	*p*-Value	Δ (Change) ρ	*p*-Value
ETDQ-7 vs. R	0.48	<0.001	0.44	<0.001	0.52	<0.001
ETDQ-7 vs. C2	−0.36	0.002	−0.33	0.004	−0.43	<0.001
ETDQ-7 vs. C2–C1	0.42	<0.001	0.37	0.001	0.47	<0.001
NOSE vs. R	0.31	0.006	0.29	0.010	0.34	0.003
NOSE vs. C2	−0.41	<0.001	−0.46	<0.001	−0.49	<0.001
NOSE vs. C2–C1	0.39	<0.001	0.35	0.002	0.41	<0.001

## Data Availability

The raw data supporting the conclusions of this article will be made available by the authors on request.

## Abbreviations

The following abbreviations are used in this manuscript:

ET	Eustachian Tube
ETD	Eustachian Tube dysfunction
NSD	Nasal septal deviation
CRS	Chronic Rhinosinusitis
FESS	Functional Endoscopic Sinus Surgery
TMM	Tubomanometry
